# Bacterial extracellular vesicles: towards realistic models for bacterial membranes in molecular interaction studies by surface plasmon resonance

**DOI:** 10.3389/fmolb.2023.1277963

**Published:** 2023-12-13

**Authors:** Maxim S. Bril’kov, Victoria Stenbakk, Martin Jakubec, Terje Vasskog, Tone Kristoffersen, Jorunn Pauline Cavanagh, Johanna U. Ericson, Johan Isaksson, Gøril Eide Flaten

**Affiliations:** ^1^ Drug Transport and Delivery Research Group, Department of Pharmacy, Faculty of Health Sciences, UiT the Arctic University of Norway, Tromsø, Norway; ^2^ Chemical Synthesis and Analysis Research Group, Department of Chemistry, Faculty of Natural Sciences and Technology, UiT the Arctic University of Norway, Tromsø, Norway; ^3^ Natural Products and Medicinal Chemistry Research Group, Department of Pharmacy, Faculty of Health Sciences, UiT the Arctic University of Norway, Tromsø, Norway; ^4^ Pediatric Research Group, Department of Clinical Medicine, Faculty of Health Sciences, UiT the Arctic University of Norway, Tromsø, Norway; ^5^ Research Group for Host Microbe Interactions, Department of Medical Biology, Faculty of Health Sciences, UiT the Arctic University of Norway, Tromsø, Norway

**Keywords:** antimicrobial resistance, outer membrane vesicles, protein-lipid interactions, membrane interactions, native barriers, *Klebsiella pneumoniae*, *Acinetobacter baumannii*, *Pseudomonas aeruginosa*

## Abstract

One way to mitigate the ongoing antimicrobial resistance crisis is to discover and develop new classes of antibiotics. As all antibiotics at some point need to either cross or just interact with the bacterial membrane, there is a need for representative models of bacterial membranes and efficient methods to characterize the interactions with novel molecules -both to generate new knowledge and to screen compound libraries. Since the bacterial cell envelope is a complex assembly of lipids, lipopolysaccharides, membrane proteins and other components, constructing relevant synthetic liposome-based models of the membrane is both difficult and expensive. We here propose to let the bacteria do the hard work for us. Bacterial extracellular vesicles (bEVs) are naturally secreted by Gram-negative and Gram-positive bacteria, playing a role in communication between bacteria, as virulence factors, molecular transport or being a part of the antimicrobial resistance mechanism. bEVs consist of the bacterial outer membrane and thus inherit many components and properties of the native outer cell envelope. In this work, we have isolated and characterized bEVs from one *Escherichia coli* mutant and three clinical strains of the ESKAPE pathogens *Klebsiella pneumoniae*, *Acinetobacter baumannii*, and *Pseudomonas aeruginosa*. The bEVs were shown to be representative models for the bacterial membrane in terms of lipid composition with speciesstrain specific variations. The bEVs were further used to probe the interactions between bEV and antimicrobial peptides (AMPs) as model compounds by Surface Plasmon Resonance (SPR) and provide proof-of-principle that bEVs can be used as an easily accessible and highly realistic model for the bacterial surface in interaction studies. This further enables direct monitoring of the effect induced by antibiotics, or the response to host-pathogen interactions.

## Introduction

The development of antimicrobial resistance (AMR) is a serious threat to public health and the main obstacle to successful treatment of infectious diseases. AMR caused 4.95 million deaths globally already in 2019 and the numbers are growing (WHO, 2014; Antimicrobial Resistance Collaborators, 2022). The antimicrobial resistant bacteria responsible for more than 15% of hospital acquired infections are classified as high priority pathogens by the World Health Organization (WHO). The Gram-negative ESKAPE (*Enterococcus faecium*, *Staphylococcus aureus*, *Klebsiella pneumoniae*, *Acinetobacter baumannii*, *Pseudomonas aeruginosa*, and *Enterobacter* spp.) strains *K*. *pneumoniae*, *A*. *baumannii*, and *P*. *aeruginosa* are on top of the list and are thus representing the strains of critical priority (WHO, 2017; Mulani et al., 2019).

To fight the ongoing AMR crisis effort is put into the discovery and optimization of new antimicrobial compounds. As most aspiring antibiotics at some point need to either cross or interact with the bacterial membrane, there is a need for cost-effective native-like models and efficient methods to characterize such interactions (Delcour, 2009).

The cell envelope of Gram-negative bacteria is a complex structure consisting of inner and outer membranes. The inner membrane is mainly composed of phospholipids, while the outer membrane is asymmetric, mainly containing phospholipids (PLs) at the inner leaflet and lipopolysaccharides (LPS) at the outer leaflet. Lipid A is the major structural unit of LPS resulting in a negative net charge. During infection, or in response to antibiotic treatment, bacteria can modify lipid A to alter the surface charge which could lead to reduced binding of cationic antimicrobials such as colistin (Olaitan et al., 2014; Llobet et al., 2015; Jeannot et al., 2017; Jasim et al., 2018; Hobby et al., 2019). The periplasmic space between the inner and outer membrane is enriched in peptidoglycans. The cell envelope is also the site for various proteins such as ion pumps and channels, receptors and membrane bound enzymes (Beveridge, 1999; Silhavy et al., 2010; Roier et al., 2016). Simple synthetic lipids in the form of liposomes, supported lipid bilayers, or nanodiscs are often utilized to investigate new antimicrobial compounds. However, they lack the intricate complexity observed in the native cell envelope (Denisov et al., 2004; Sezgin and Schwille, 2012; Akbarzadeh et al., 2013; Scheidelaar et al., 2015; Denisov et al., 2019; Jackman and Cho, 2020; Bailey-Hytholt et al., 2021). Incorporation of all native outer membrane components into synthetic model liposomes is a possible but laborious and expensive process (Prenner et al., 1999; Pirc and Ulrih, 2015; Elderdfi and Sikorski, 2018; Paulowski et al., 2020; Jakubec et al., 2021). There is thus a need for affordable but relevant model systems that can accurately mimic the bacterial membrane.

Bacterial extracellular vesicles (bEVs) are formed mainly from outer membranes and maintain similar composition containing PLs, LPS and OM-proteins, and thus, can represent naturally assembled nano-models of the outer membranes (Haurat et al., 2015; Roier et al., 2016). For bacteria, the bEVs play an extensive role in physiological and pathogenic processes in, for example, molecular transport, biofilm formation, antibiotic resistance and cell-cell communication. As a part of the bacterial defense mechanism, bEVs serve as a decoy, absorbing and neutralizing membrane active agents, including bacteriophages. bEVs can also be a central part of a disposal mechanism by which bacteria can get rid of damaged/affected membranes (Manning and Kuehn, 2011; Balhuizen et al., 2021). EVs are ideal candidates to serve as the outer membrane model for the bacterial strain it is produced from.

The aim of this work was to explore the applicability of native bEVs as bacterial membrane models in interaction studies. To achieve this, bEVs secreted by overproducing *E. coli* Δ*tolA* mutant (Reimer et al., 2021), LPS deficient *E*. *coli* NR698 strain (Ruiz et al., 2005; Krüger et al., 2019) and three clinical multi-resistant strains of critical importance *A*. *baumannii* K47-42, *P*. *aeruginosa* K34-7 and *K*. *pneumoniae* K47-25 (Paulsen et al., 2021) were isolated and characterized. The clinical strains were previously shown to be beta-lactam resistant caused by beta-lactamase production (Paulsen et al., 2021). The *K*. *pneumoniae* and *A*. *baumannii* strains are also colistin resistant, being able to modify lipid A in the outer membrane (Paulsen et al., 2021). The kinetic rates of interaction between the bEVs and membrane active compounds were measured by surface plasmon resonance (SPR). Antimicrobial peptides (AMPs) are receiving increasing attention in the fight against AMR, as their different mode of actions lead to destabilization of bacterial membranes and bacterial death (Czaplewski et al., 2016; Mahlapuu et al., 2016; Ageitos et al., 2017; Kumar et al., 2018). A test panel of five cyclic AMPs with various distributions of charged and hydrophobic amino acids, known to interact with bacterial membranes was used (Junkes et al., 2008; Bagheri, 2010; Gopal et al., 2012; Finger et al., 2015; Rainsford et al., 2022; Jakubec et al., 2023). This set of peptides was originally designed to study how the peptide charge distribution affects membrane interactions and was used by our lab to explore kinetics of binding towards synthetic liposomes, nanodiscs and to a series of *E*. *coli* strains (Rainsford et al., 2022; Jakubec et al., 2023).

## Results and discussions

In this work we aimed to apply bEVs as a relevant model of the native bacterial membrane for interaction studies by SPR. Antimicrobial peptides were used as test compounds to establish proof of principle. An *E*. *coli* strain with a deletion of the *tolA* gene, which results in higher bEV yields (Reimer et al., 2021), was used as a starting point to establish the production- and isolation protocols. The LPS deficient *E*. *coli* strain NR698 (Ruiz et al., 2005; Krüger et al., 2019) as well as clinical multidrug resistant strains of *A*. *baumannii*, *P*. *aeruginosa*, and *K*. *pneumoniae* were further selected. The latter belong to the group of critical priority ESKAPE pathogens listed by WHO (WHO, 2017). First, bEVs were characterized in terms of morphology and lipid composition and compared to bacterial lipid isolates from the same strains. Then bEVs were directly applied to SPR measurements of interactions with the selected test compounds. In addition to the bEVs, liposomes were prepared from lipids isolated from *E*. *coli* Δ*tolA* and used as control vesicles in this study.

### Characterization of native bEVs

#### Morphology and zeta potential of isolated bEVs

**FIGURE 1:**
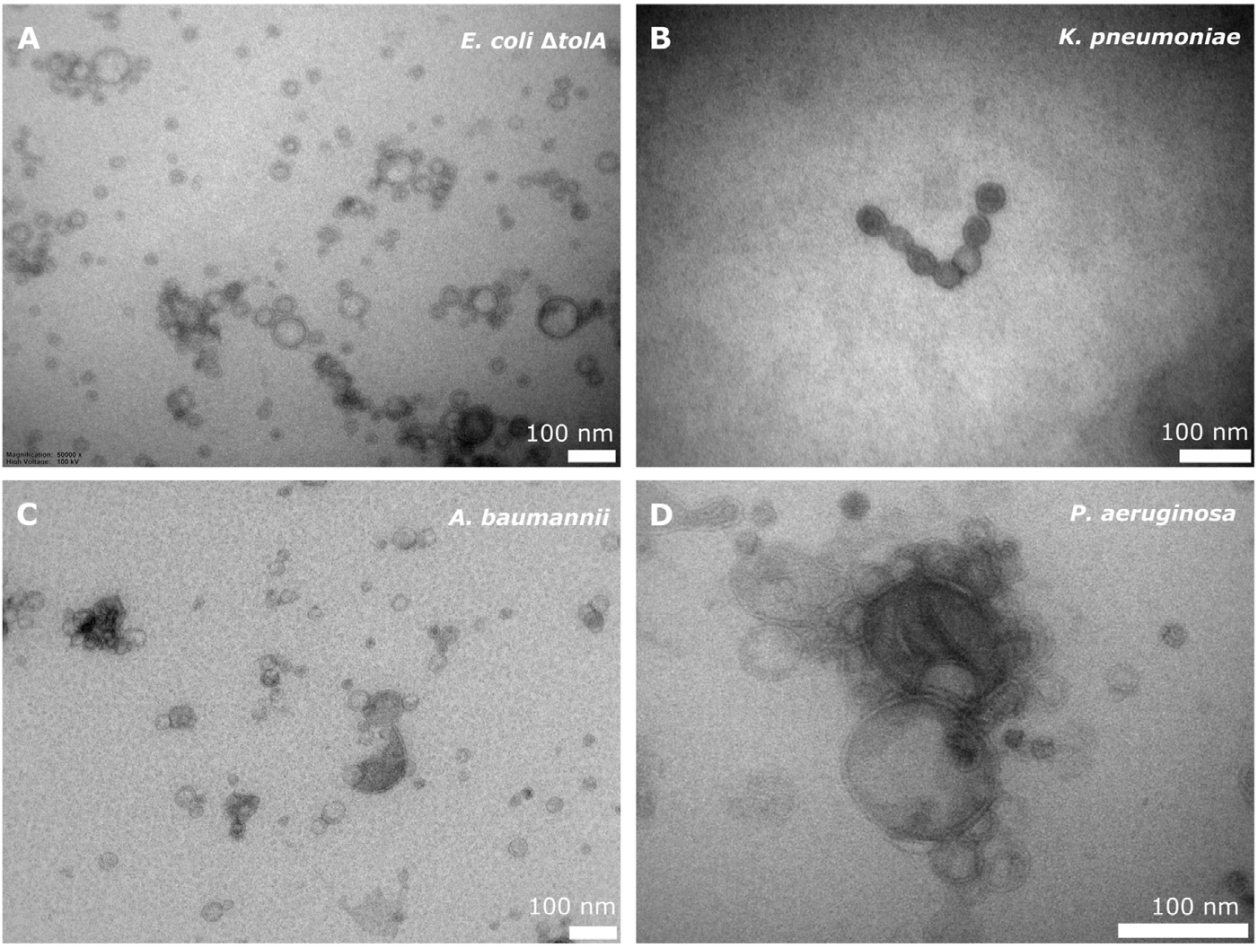
TEM micrograph of bEVs secreted by *E. coli* ΔtolA **(A)**, *K. pneumoniae* **(B)**, *A. baumannii* **(C)** (50,000 x magnification), and *P. aeruginosa* **(D)** (80,000 x magnification). 100 nm scalebar is presented at the bottom right corner of each micrograph.

All strains, except the LPS deficient *E*. *coli* NR698, produced bEVs in sufficient amounts and quality. Isolated bEVs were visualized by transmission electron microscopy (TEM) to assess morphology and sample quality. Figure 1A–D shows micrographs of representative samples of bEVs from all strains, except *E*. *coli* NR698. The samples appear to be free from contamination with only minor traces of cell debris, confirming the successful isolation of the bEVs. All vesicles observed were spherical with a visible double layer. The bEVs from *P*. *aeruginosa* strain seemed aggregated (Figure 1D), a feature commonly observed during the study of this strain. For the LPS deficient *E*. *coli* NR698 strain, although some bEVs were present, the sample was dominated by deformed bEVs (Supplementary Figure S1). The strain has mutations causing deficiency of LPS in the outer membrane, possibly resulting in a higher content of phospholipids (Ruiz et al., 2005; Krüger et al., 2019). This sample represents one limitation of bEVs application and experimental design regarding genetic alterations affecting the properties of the outer membrane. Due to the inadequate quality of the isolated bEVs, this strain was not further pursued.

**FIGURE 2:**
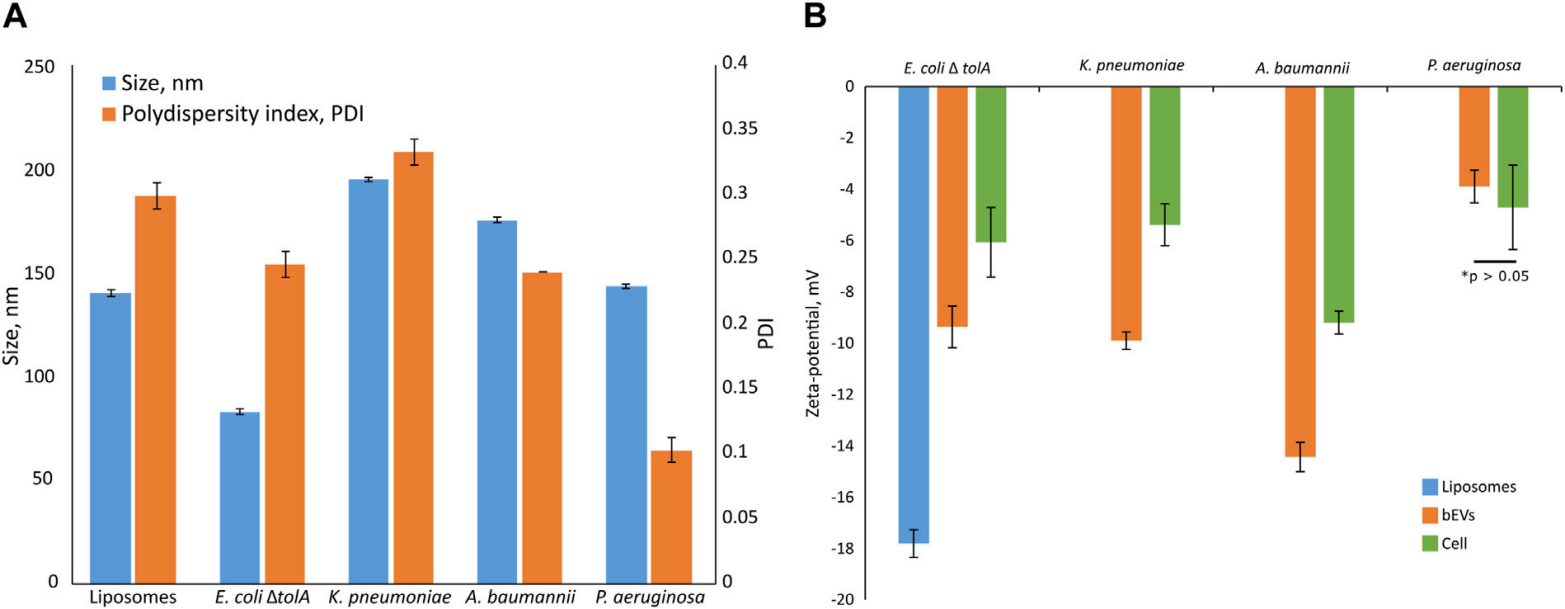
Summary of size and polydispersity index (PDI) **(A)** and zeta-potential measurements **(B)** for isolated bEVs in comparison to zeta-potential of the bacterial cells. The sample of liposomes prepared from *E. coli* ΔtolA lipid isolate is also included. Samples were held in 10 mM TRIS buffer (100 mM NaCl and 5 mM KCl, pH 8.0) at 25°C. All samples were analyzed in triplicates.

The average vesicle diameter was determined to be in the range from 194.8 ± 1.0 nm for *K*. *pneumoniae*, 175.0 ± 1.3 nm for *A*. *baumannii* and 143.3 ± 1.1 nm for *P*. *aeruginosa*, to 82.7 ± 1.3 nm for *E*. *coli* Δ*tolA* (Figure 2A). When comparing the zeta potentials of bEVs and bacterial cells (Figure 2B; Supplementary Table S1), the values are found to be significantly lower for bEVs in all the cases except *P*. *aeruginosa*, where no significant difference was observed (*t*-test, *p* > 0.05). The lowest zeta potential of −14.4 ± 0.6 mV was measured for bEVs from *A*. *baumannii*. bEVs from *K*. *pneumoniae* and *E*. *coli* Δ*tolA* showed similar values for zeta potentials of −9.9 ± 0.3 mV and −9.4 ± 0.8 mV, respectively. The zeta potential of bEVs from *P*. *aeruginosa* was measured to −3.90 ± 0.6 mV, which suggests a less stable dispersion due to less repulsion between the vesicles, and could, thus, explain the aggregation observed (Figure 1D). Liposomes prepared from *E*. *coli* Δ*tolA* lipid isolate were used as a control in our studies and were also characterized according to size distribution and zeta potential. They showed an average size of 139.9 ± 1.6 nm and had a significantly lower zeta potential, −17.8 ± 0.5 mV, compared to both bEVs produced by the same bacteria, and the whole bacteria itself. This is an indication that the bEVs better represent the bacterial surface than liposomes prepared from lipid isolates. The size of the bEVs isolated from *E*. *coli* Δ*tolA* was reanalyzed after 1 month of storage at 4°C. No significant change in size was observed, suggesting that the bEVs were stable for at least 1 month. All samples were analyzed in triplicates.

#### Lipid composition of native bacterial membranes

The compositions of the bacteria lipid isolates were quantified by ^31^P NMR, and the qualitative pattern was acquired by MS. The corresponding pattern was subsequently acquired for lipid isolates of the corresponding secreted bEVs from the same strains.

**FIGURE 3:**
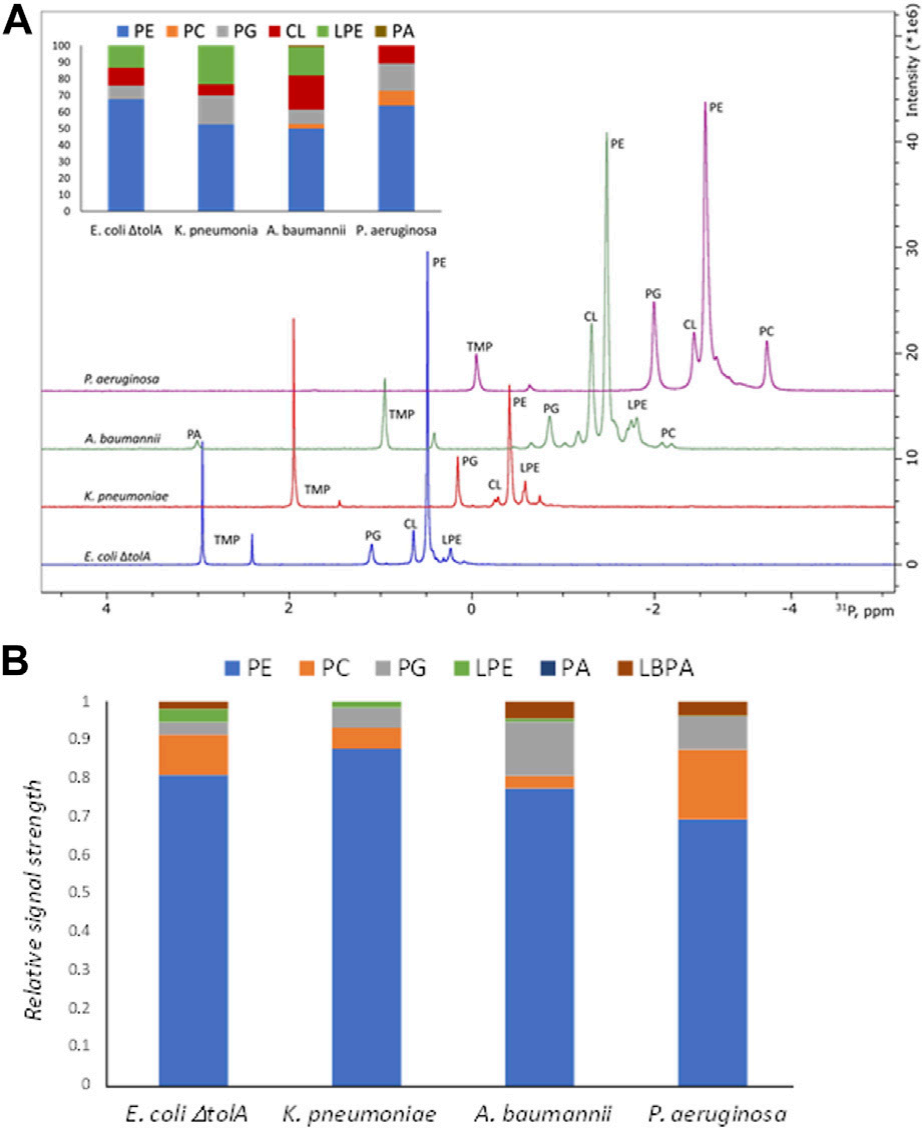
Summary of lipidomics analysis of bacterial lipid isolates by ^31^P NMR **(A)** and Mass Spectrometry **(B)**. Intersection in the panel A shows quantified composition of the lipids in %. Traces of the NMR spectra were shifted up and right to ease the display. Here PE, phosphatidylethanolamine; PC, phosphatidylcholine; PG, phosphatidylglycerol; LPE, lyso-phosphatidylethanolamine; CL, cardiolipin; PA, phosphatidic acid; LBPA, lysobisphosphatidic acid.

For gram negative bacteria in general, the bacterial membrane is expected to be dominated by Phosphatidylethanolamine (PE) supplemented by the negative phospholipids Phosphatidylglycerol (PG) and cardiolipin (CL). The results from the ^31^P NMR studies on the whole bacterial lipid isolates are shown in Figure 3A and confirmed the expected general pattern that PE was the dominant phospholipid, ranging from 50%–52% (for *A*. *baumanii* and *K*. *pneumoniae*) up to 64%–68% (for *P*. *aeruginosa* and *E*. *coli* Δ*tolA*) of the total phospholipid content. The CL content was found to be 7% for *K*. *pneumoniae*, 10%–11% for *P*. *aeruginosa* and *E*. *coli* Δ*tolA* and up to 21% for *A*. *baumannii*. Lyso-phosphatidylethanolamine (LPE) was determined to be at 24%, 17% and 13% levels for *K*. *pneumoniae*, *A*. *baumannii* and *E*. *coli* Δ*tolA* respectively, while no LPE was detected in *P*. *aeruginosa*. Phosphatidylcholine (PC) contributed to 3% and 9% in *A*. *baumannii* and *P*. *aeruginosa* respectively, while it was not detected in *E*. *coli* Δ*tolA* and *K*. *pneumoniae*. PG contributed to 9% of the total phospholipids in *E*. *coli* Δ*tolA* and *A*. *baumannii*, and up to 17% for *K*. *pneumoniae* and *P*. *aeruginosa*. In conclusion, the expected pattern for gram negative bacteria was confirmed, where PE lipid was the major lipid of the studied bacterial membranes, together with a handful of other phospholipids in smaller quantities, in agreement with previous reports (Wassef, 1976; Sohlenkamp and Geiger, 2016; Deschamps et al., 2021; Tao et al., 2021).

**FIGURE 4:**
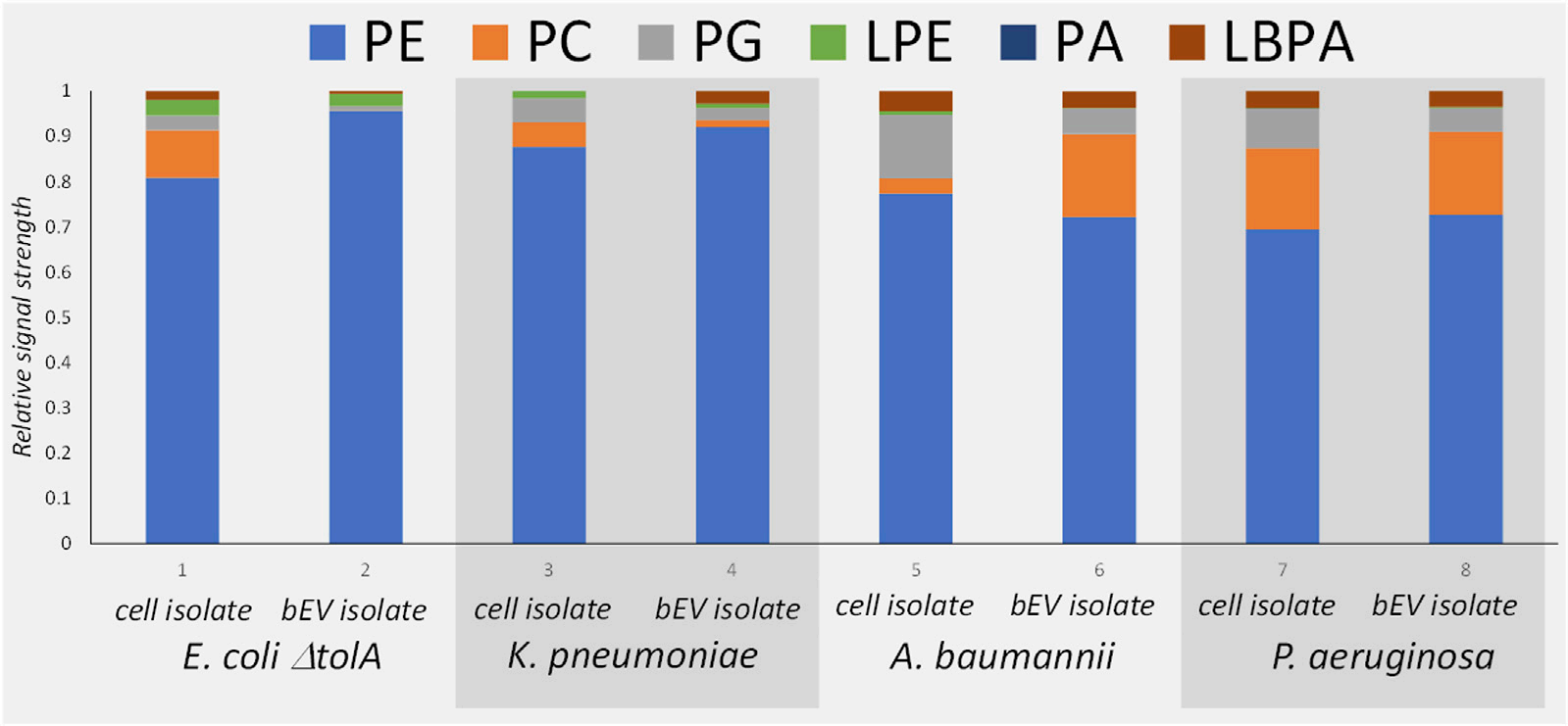
Mass Spectrometry patterns of the phospholipid compositions of the bEVs in comparison to the lipid isolates of corresponding strains: *E. coli* ΔtolA, *K. pneumoniae*, *A. baumannii* and *P. aeruginosa*. Here PE, phosphatidylethanolamine; PC, phosphatidylcholine; PG, phosphatidylglycerol; LPE, lyso-phosphatidylethanolamine; PA, phosphatidic acid; LBPA, lysobisphosphatidic acid.

The MS analysis (Figure 3B) confirmed the presence of the same major lipid classes as in the NMR. Although the available MS approach only allowed for non-quantitative comparison of the relative signal within each sample, the results are in qualitative agreement with the ^31^P NMR in that PE was the dominant lipid class in all samples (Figure 4). The relative abundance of PC is the highest for bEVs from *P*. *aeruginosa* and A. baumannii, and the observation was consistent with ^31^P NMR. While PC is commonly found in *P*. *aeruginosa*, this phospholipid is normally not present in the bacterial membrane of *E*. *coli* strains (Sohlenkamp and Geiger, 2016; Deschamps et al., 2021). What is presented is however the relative signal, and a high signal of PC does not only reflect the concentration of the lipid but also the ability to ionize. Further, only PC with 18°C-atoms in the fatty acid chains were detected, which is not what is expected from a naturally occurring class of lipids.

Bacteria are typically missing the biosynthetic pathways for atypical lipids, but they can sometimes utilize fatty acids, phospholipids or their precursors available in the environment to adapt and, for example, evade the host immune defense system (Heung et al., 2006; Hobby et al., 2019). The strains in this work were cultured in rich lysogeny broth (LB) medium, which is based on yeast extracts (Alvarez and Steinbuchel, 2002; Montefusco et al., 2014). Phospholipid analysis of LB media by ^31^P NMR revealed only trace amounts of PE and PA lipids (Supplementary Figure S2), thus it is possible that LB media is a source of minor PA or LBPA contaminations. We therefore concluded that the growth media did not significantly influence the phospholipid composition of the bacteria or the vesicles.

In general, quantitative comparison between NMR (quantitative relative abundance) and MS (non-quantitative relative abundance) were interpreted conservatively. The higher sensitivity of MS allowed us to get data also from the lipid isolates of the bEVs, and to compare the pattern to that of the total lipid isolates of the same bacteria.

The comparison between the lipid isolates from the whole cells and the corresponding bEVs (Figure 4) provided a qualitatively good match between the lipid patterns of the bEVs and the parent bacteria. Since the lipid isolate contains lipids from the whole bacteria not just the membrane, the lipid isolate from the bEVs are not expected to be identical to the mother bacteria but contain the same pattern of the most dominant lipid classes as the bacteria. The results again confirmed that PE is the dominating lipid, and over all the same minor lipids are identified. The main deviation was observed for *A*. *baumannii*, which showed different distributions of PC between the bacterial extracellular vesicles and the whole cell isolate, having more PC in the outer membrane (Figure 4). The MS approach also allowed fatty acid composition analysis of the detected lipid species, but was limited to the PE phospholipid as it is the most dominant species in all samples. Out of the detected fatty acids, the hits with a relative abundance higher than 1% were selected for analysis (Supplementary Table S2). Mono-unsaturated fatty acids dominated in all samples. The top hits with relative abundance above 10% are mono-unsaturated or saturated fatty acids with a chain length of 16 or 18 carbon atoms in different combinations. The results also suggested higher preferences of *K*. *pneumoniae* for shorter fatty acid chains with 14 carbons, or chains with odd number of carbons (17 or 19) instead of 18 carbons. For the *E*. *coli* Δ*tolA* sample, a fatty acid with 20 carbons and 4 double bonds was detected. When comparing the bEVs’ fatty acid composition to the lipids isolated from the whole bacteria it was observed that *E*. *coli* Δ*tolA* had similar lipid compositions in both the bEVs and the native cell, while for the ESKAPE strains some differences could be observed. In particular this was evident for *K*. *pneumoniae* where the bEVs to a higher extent contained even numbered fatty acid chains (e.g., PE 16:0_16:1) compared to the whole bacteria where the PE 16:0_17:1 and PE 16:0_19:1 were the most prevalent. This could affect its lipid phase and physical properties like permeability, flexibility and fluidity. The dynamic nature of membrane lipid metabolism is another factor that can allow bacteria to survive antimicrobials by modifying lipids, fatty acids distribution or their whole lipid composition (Staubitz et al., 2004; Shireen et al., 2012; Schenk et al., 2015; Han et al., 2018; Bisignano et al., 2019; Tao et al., 2021).

In conclusion, the bEVs are shown to broadly inherit the lipid composition of the parent bacterial membrane, including species specific variations. Thus, they show potential to be an elegant model system for targeted studies, carrying authentic bacterial outer membrane in a highly stable and reasonably sized nano-construct. As such they can be seen as a much more relevant mimic of the bacterial cell surface compared to synthetic liposome membranes.

### Interaction studies by SPR

The potential to use bEVs as models resembling the bacterial membrane in interaction studies was explored. As proof-of-principle, the interactions between bEVs and AMPs were measured using SPR.

**FIGURE 5:**
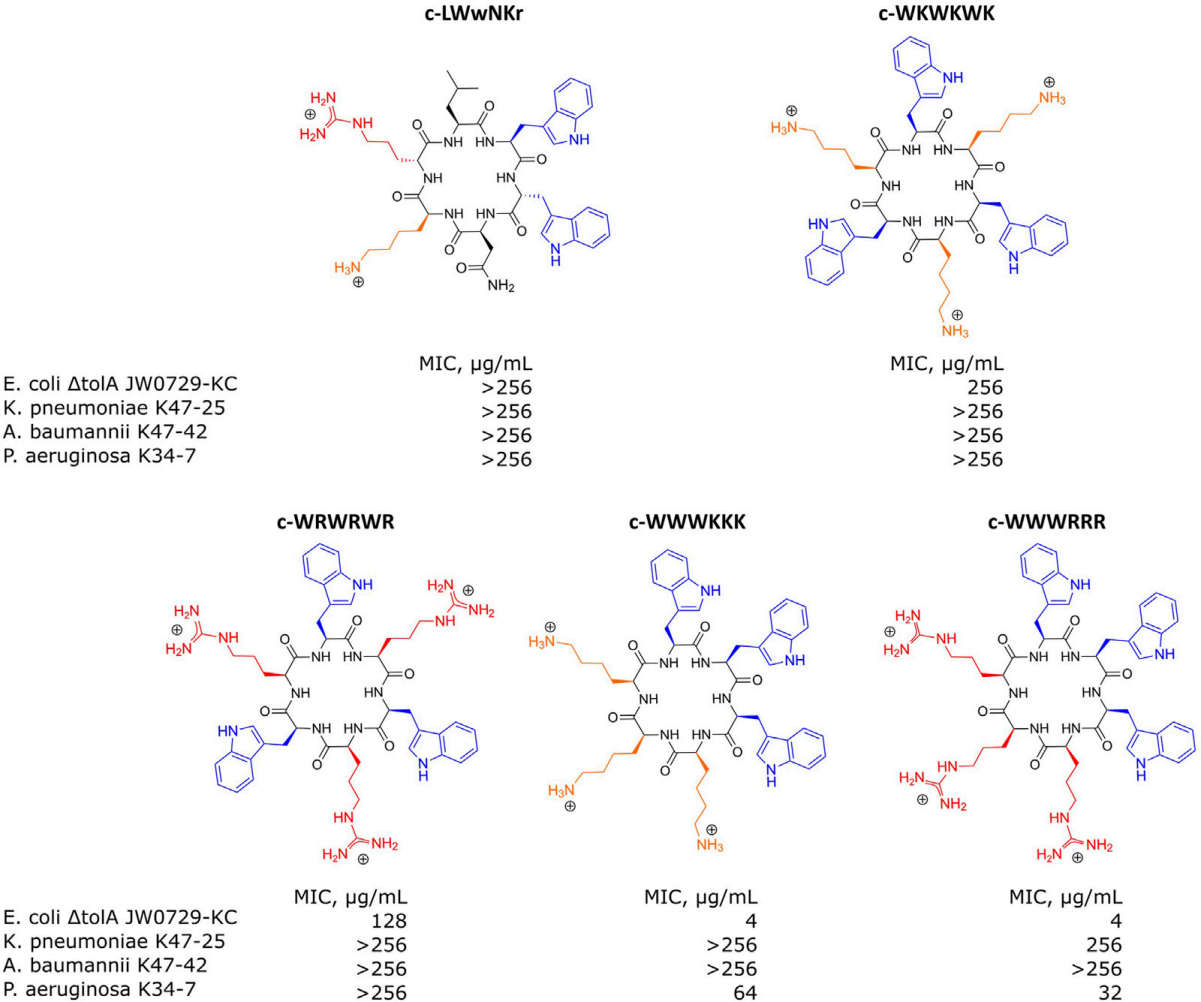
Structures of antimicrobial peptides used in this study and their minimal inhibitory concentrations (MIC) values determined for *E. coli* ΔtolA JW0729-KC, *K. pneumoniae* K47-25, *A. baumannii* K47-42 and *P. aeruginosa* K34-7. Hydrophobic Tryptophans highlighted in blue, positively charged Arginines and Lysines are highlighted in red and orange correspondingly.

For the SPR analysis, samples of either bEVs or liposomes were immobilized at the surface of L1 chips, and the dilution series of the antimicrobial peptides were run over the layers, measuring the response. A set of synthetic cyclic AMPs with alternating- or clumped distribution of charged (Lysine or Arginine) and hydrophobic (Tryptophan) amino acids was used: c-WKWKWK, c-WRWRWR, c-WWWKKK and c-WWWRRR (Figure 5). The set was selected as a useful chemical model to study the effects of charge distribution on the interactions. The peptide c-LWwNKr (Figure 5) was included in the study as a control that is chemically similar, but without antimicrobial activity (MIC > 256 μg/mL) (Junkes et al., 2008; Bagheri, 2010; Gopal et al., 2012; Finger et al., 2015; Rainsford et al., 2022; Jakubec et al., 2023).

**FIGURE 6:**
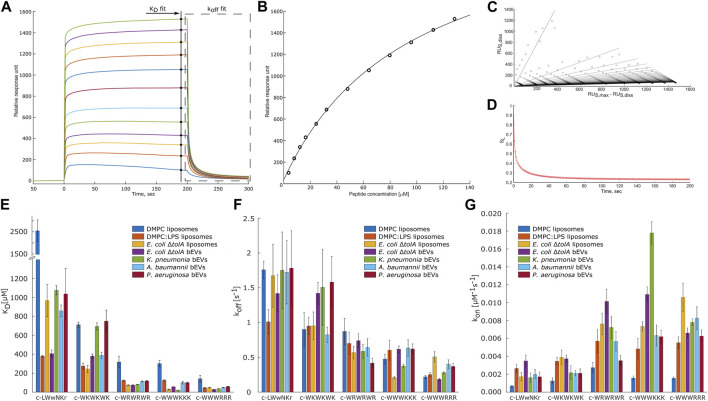
Overview panel of the SPR method and results. **(A)** – example of typical raw response signals collected during a run. Marked data points used for fitting to models to obtain K_D_ **(B)** and k_off_ values. To obtain k_off_ values the raw data of the marked region first was linearized **(C)** and then a model was fitted into in-time changing linear coefficients **(D)**. **(B–D)** represent typical data fit plots for our work. **(E,F)** summarize measured K_D_ and k_off_ values. **(G)** shows calculated k_on_ values. Three parallels were performed for each combination. Values are available in Supplementary Tables S3–S5 in Supplementary Material. T-test statistical analysis of the data is summarized in Supplementary Table S6 in Supplementary Material. Results for DMPC and DMPC:LPS (90:10) are reproduced from Jakubec et al. (2023).

Interactions were analyzed using the model described by (Figueira et al., 2017). By this, the dissociation constant K_D_ and the disassociation rate k_off_ were obtained. These constants allowed us to calculate k_on_ rate, using Eq. 4. Figures 6A–D present examples of the raw response curves obtained during a run and the analytical processing steps of data fitting and analysis. The results for all samples are summarized in Figures 6E–G; Supplementary Tables S3–S5.

MIC determinations for each of the peptides against the bacterial strains used in this study are shown in Figure 5. The non-resistant *E*. *coli* Δ*tolA* displayed strong binding (low K_D_) and high antimicrobial activity (MIC = 4 μg/mL for both c-WWWKKK and c-WWWRRR). However, for the clinical strains none of the peptides showed any activity (MIC ≥ 256 μg/mL), except c-WWWKKK and c-WWWRRR that were inhibiting growth of *P*. *aeruginosa* at ≥64 μg/mL and ≥32 μg/mL, respectively. This does not directly reflect the fact that 3 out of the 4 antimicrobially active peptides bind as efficiently to the surface of the bEVs from resistant strains as to the bEVs from susceptible strains. The c-WKWKWK displayed a weaker interaction with the bEVs from *K*. *pneumoniae* and *P*. *aeruginosa* while the control peptide c-LWwNKr showed no antimicrobial activity as expected.

#### Quantification of the non-lipid components’ contribution

Any detailed analysis of the non-lipid contents of the bEVs have been out of the scope of this work. However, comparing the observed interactions of the peptides with *E*. *coli* Δ*tolA* bEVs and liposomes assembled from the lipid isolate of the same strain gives an indication of how the kinetic barrier imposed by LPS together with membrane proteins and other native components affects the interaction. For lysine-containing peptides, both the k_off_ and k_on_ rates were positively affected for the interactions with the bEVs, but the effect was more pronounced for k_off_, resulting in an overall increase in K_D._ A corresponding increase in the k_on_ and k_off_ rates for the lysine-containing peptides was also observed in the recently published comparison between homogeneous DMPC liposomes and DMPC liposomes containing 10% LPS (Jakubec et al., 2023) (results included in Figures 6E–G). In this case the balance was however different, resulting in slightly improved K_D_ and K_p_.

The opposite picture is observed for the c-WWWRRR peptide, where LPS and other components decreased both the k_off_ and k_on_ rates, shifting the balance towards decreased dissociation. In the recent study with dimyristoylphosphatidylcholine (DMPC) liposomes, LPS similarly improved K_D_, but in that case, it was driven by a significant increase of the k_on_ rate (Jakubec et al., 2023). This peptide demonstrated the strongest binding of all tested peptides, and had the lowest MIC for *E*. *coli* Δ*tolA*. For the control peptide, the native bEV mostly contributed to an increase in association rate, k_on_, and a slight decrease in dissociation rate. The resulting change in the K_D_ for this sample was the most prominent among the studied peptides. This observation was fully consistent with the trends in the synthetic DMPC/DMPC + LPS system, suggesting that LPS is likely responsible for the stronger binding of c-WWWRRR also in the bEV system.

#### Interactions of bEVs produced by ESKAPE strains

The overarching focus was to isolate and characterize bEVs from resistant clinical strains of ESKAPE pathogens, and to explore their applicability in interaction studies with antimicrobial model compounds. Comparison of the results from the interaction studies with bEVs to those with liposomes made from *E*. *coli* lipid isolate showed that the trend is consistent, with the clumped peptides being the strongest binders and the control peptide and c-WKWKWK being the weakest, reflected by both K_D_ and k_off_ (Figures 6E–G). There are, however, notable peculiarities in the results. The c-WWWKKK peptide was the peptide in this study that had the highest affinity for bEVs from *K*. *pneumoniae* and was even binding stronger to the BEVs than to the *E*. *coli* lipid isolate liposomes. The bEVs from *K*. *pneumoniae* had a prominent positive effect on the k_on_ rate and only a slight increase in the k_off_ rate, resulting in an approximately 5-fold lower K_D_ compared to the K_D_ for other clinical strains. The outer membrane of *A*. *baumannii* and *P*. *aeruginosa* had on the other hand a negative effect on the k_off_ rate and K_D_ for this peptide.

The results for the control peptide, c-LWwNKr, displayed a strong preference for the outer membrane of *E*. *coli* Δ*tolA* over the lipid isolate liposomes. A similar increase was observed in DMPC + LPS liposomes compared to pure DMPC liposomes, and in both cases this increase is driven by an increase in k_on_. Even though this peptide is inactive, it exhibits this apparent affinity for LPS. This increase was not observed in the resistant strains. However, similar trends in interaction of resistant strains and the c-WKWKWK peptide could be found. Here the k_on_ rate (Figure 6E) was reduced similarly for bEVs from the resistant strains compared to the *E*. *coli* lipid isolate liposomes, while for the bEVs from the non-resistant *E*. *coli* Δ*tolA* there was no statistically significant change in k_on_. The k_off_ rate (Figure 6F) was slightly decreased for *A*. *baumannii* while significantly increasing for the other strains, including the susceptible *E*. *coli* Δ*tolA*. Thus, as a result the K_D_ values (Figure 6G) of bEVs from *A*. *baumannii* is similar with bEVs from *E*. *coli* Δ*tolA*, while for bEVs from *K*. *pneumoniae* and *P*. *aeruginosa* a twofold increase in K_D_ was displayed.

For the interactions with the c-WRWRWR peptide, the bEVs from the resistant strains displayed a decrease in k_on_ rates compared to the *E*. *coli* lipid isolate liposomes, especially for *A*. *baumannii and P*. *aeruginosa*. In contrast, for the susceptible *E*. *coli* Δ*tolA*, the k_on_ rate instead increased for this peptide compared to the lipid isolate liposomes. The c-WWWRRR peptide showed a consistent behavior for all samples, both k_on_ and k_off_ rates being slower compared to the lipid isolate, resulting in only minor changes in K_D_.

The overall trend when comparing the peptides to each other is, however, that the peptides that have a strong affinity for the *E*. *coli* lipid isolate and the bEVs of the susceptible strain, also show a strong affinity for the bEVs from the resistant strains. The only exception is the c-WKWKWK peptide with a moderate affinity, which displayed a weaker affinity for two out of the three resistant strains.

bEVs from ESKAPE pathogens further appear to have inherited the strain specific properties of the outer membranes, reflecting the native surface of the bacteria. Their different properties resulted in corresponding differences in their interactions with AMPs measured by SPR. Further, comparison to liposomes made from whole bacteria lipid isolates allowed us to distinguish between the contributions from the lipids and the other outer membrane components, presumably dominated by LPS. The results demonstrated that the affinity of the AMPs to ESKAPE strains did not directly reflect their antimicrobial activity. The strongest binder was inactive towards the resistant strains, while being active in non-resistant strain with comparable binding parameters. Some potential explanations why there are no close correlations between the binding affinity and the antimicrobial effect could be that the resistant bacteria either maintain the membrane integrity upon association, or the bacteria have a mechanism to efficiently deactivate the AMPs (Manning and Kuehn, 2011; Balhuizen et al., 2021). Another possible explanation why the peptides would bind but not cause bacterial death is that the peptide did not manage to penetrate the kinetic conditions set by LPS and its modifications (Olaitan et al., 2014; Llobet et al., 2015; Jeannot et al., 2017).

## Conclusion

The traditionally used synthetic membrane models for the bacterial membrane cannot effectively reconstruct the full complexity of native bacterial membranes. Herein, we have for the first time shown that the bacteria can do the hard work for us and produce sufficiently large quantities of native bEVs that enable interaction studies by SPR between compounds of interest and authentic bacterial outer membranes. The isolated bEVs were found to broadly inherit the lipid composition of the parent bacterial membrane, including species specific variations of the outer membrane composition, which in turn dictated the kinetic rates of the interaction processes. bEVs can thus be seen as a much more relevant mimic of the bacterial cell surface compared to synthetic liposome membranes.

Thus, we have provided proof of principle that native bEVs produced by clinical strains of Gram-negative ESKAPE strains, carrying specific antimicrobial resistance genes, can be used as tools to study processes on the bacterial surface. This sets the conditions to enable direct quantification of, for example, the effect of induction by antibiotics, or the response to host-pathogen interactions.

## Materials and methods

### Materials

All common chemicals and solvents were purchased from Sigma Aldrich (Merk KGaA, Darmstadt, Germany). *Escherichia coli* Δ*tolA* strain was obtained from National BioResource Project (NIG, Japan): *E*. *coli* (Keio Collection JW0729-KC). Strains of clinical isolates of multi-drug resistant *K*. *pneumoniae* K47-25, *A*. *baumannii* K47-42 *and P*. *aeruginosa* K34-7 strains were provided by The Norwegian National Advisory Unit on Detection of Antimicrobial Resistance (K-res), University Hospital of Northern-Norway (UNN). The peptides c-WKWKWK, c-WRWRWR, c-WWWKKK, c-WWWRRR and c-LWwNKr were synthesized as previously reported in (Rainsford et al., 2022; Jakubec et al., 2023). PhosSTOP tablets were purchased from Roche via Sigma Aldrich. Lipids were purchased from Avanti Lipids via Sigma Aldrich.

### Minimum inhibitory concentration (MIC) determination

MIC for all test peptides was determined following EUCAST clinical standard (Hasselmann, 2003). Briefly, 3–4 colonies were picked by a cotton swab from freshly plated cultures on agar and resuspended in sterile saline solution. Turbidity of inoculum was adjusted to 0.5 McFarland standard units prior to ×100 dilution in Mueller-Hinton broth. Antimicrobial peptides were dissolved in the broth at the stock concentration of 512 μg/mL. MIC was determined in series of dilutions of antimicrobial peptides at concentrations ranging from 256 to 2 μg/mL in microdilution plates, keeping concentration of bacterial cells at constant. Positive control of bacterial culture diluted with broth and negative control of blank broth were included for each bacterial strain. The MICs were determined after incubation of the plates at 37°C for 18 h and red as the concentration of antimicrobial peptide at the next to the last well where bacterial growth was detected.

### Bacterial extracellular vesicles isolation

Bacterial cultures were grown in lysogeny broth (LB) media at 37°C at 120 rpm until stationary growth phase for 16 h and harvested by centrifugation at 10,000 × g for 20 min. For further lipidomic analysis the bacterial pellet was lysed by immediate resuspension in 5 mL of TRIS buffer (10 mM TRIS, 100 mM NaCl and 5 mM KCl, pH 8.0) supplemented with 1 tablet of PhosSTOP, 2 mg/mL of 2-butoxyphenylboronic acid (BPBA), and 500 µL of mixture of mutanolysin (50 µg), lysozyme (10 mg), RNAase (200 µg) per 1 mL of glycerol/PBS 1:1 (the stock stored at −80°C). The mixture was left overnight at room temperature on a soft shaker and then frozen at −80°C before being freeze dried.

For isolation of BEVs the collected supernatant was filtered through 0.45 µm PES bottle-top vacuum filters and further upconcentrated to approx. 50–100 mL using Sartorius VivaFlow 200 30,000 PES membrane according to the instruction manual. Concentrate was filtered through 0.22 µm filters using a syringe, and then was ultracentrifuged (Beckman Coulter, Optima XPN-100) at 30,000 rpm for 2 h. The supernatant was discarded, and the pellet was resuspended in TRIS buffer, following another run of centrifugation at 30,000 rpm for 2 h. The remaining pellet, which contains the outer membrane vesicles, was finally resuspended in 300 µL TRIS buffer and inspected by negative-stain electron microscopy to assess the quality of the samples and analyzed on Zetasizer (see below). Protein concentration was used as a reference for the samples and was measured by Bradford protein assay from Bio-Rad.

### Lipids isolation

To isolate lipids a modified Bligh and Dyer method was used (Bligh and Dyer, 1959; Furse et al., 2017). Briefly, lysed bacterial pellet was freeze dried for 48 h after being treated with the enzymatic mixture as described above. The freeze-dried material was mixed with 10–15 mL of dichloromethane/methanol mixture (2:1), properly vortexed and centrifuged (6,000 × g for 10 min). The organic solution was decanted to a separation funnel and the remaining pellet was mixed with another 10–15 mL of the same dichloromethane/methanol mixture. After vortexing the dispersion was transferred to the same separation funnel. The same volume of 5% NaCl solution in water was added to the funnel. The content of the funnel was gently mixed, and the mixture was left for the phase separation. After separation, the bottom organic phase was collected, and the remaining water phase was washed by addition of the same volume of dichloromethane/methanol mixture. Again, the mixture was allowed to form the phases and the bottom organic one was collected and combined with the previously collected fraction. The collected pooled organic mixture was filtered through filter paper and evaporated on a rotary evaporator. The resulting lipid film of *E*. *coli* Δ*tolA* was used to prepare liposomes for SPR analysis (by hydration with TRIS buffer, see below). Otherwise, the dried lipid films were redissolved in smaller volume of isopropanol and transferred to a smaller pre-weighted glass container through a cotton filter. After drying the lipid isolates under a nitrogen stream the resulting lipids were weighed. These isolates were used to prepare samples for ^31^P NMR analysis by dissolving in CUBO solvent or for MS analysis by dissolving in isopropanol (see below).

Lipid isolation from bEVs was performed in a similar fashion but with a simpler procedure. Freeze dried bEVs were resuspended in 3–5 mL of 2:1 mixture of dichloromethane and methanol, sonicated for 2 min of 2 s on/8 s off cycles on ice using probe sonicator (Ultrasonic processor 500 W, Sigma Aldrich, United States). 3–5 mL of 5% NaCl was added to the samples and after thorough vortexing the phases were separated by centrifugation at 3,000 × g for 5 min. The organic solution was transferred to a clean glass vial with glass pipet. The remaining aqueous phase was mixed with another 3–5 mL of the same dichloromethane/methanol mixture, vortexed and centrifuged. After separation, the organic phases were pooled together into the glass vial and evaporated under nitrogen. Dried isolates were redissolved in a smaller volume of isopropanol and transferred into a smaller pre-weighted glass container through a cotton filter. After drying under nitrogen, the lipid isolates were weighed. The samples were dissolved in isopropanol for mass spectrometry analysis.

### Mass spectrometry

All lipid samples were dissolved in isopropanol at 1 mg/mL concentration. Identification and relative quantification of lipids were done on a Thermo IdX Tribrid Orbitrap MS with a Thermo Vanquish UPLC. Separation was done on a Waters Acquity Premier BEH C18 1.7 um 2.1 × 100 mm column with gradient elution. Mobile phase A consisted of 50% ACN in MilliQ-water and mobile phase B of 47.5% ACN, 47.5% isopropanol and 5% MilliQ-water. Both mobile phases were buffered with 1 mM ammonium formate and added formic acid to pH 3. The gradient started with 30% B, followed by a linear increase to 75% B at 20 min, again followed by a linear increase to 95% B at 25 min, which were held for 5 min, giving a total analysis time of 30 min. The injection volume was 2 μL, the column temperature 60°C, the flow rate 0.6 mL/min, and the sample compartment was held at room temperature to avoid precipitation of lipids. Identification of lipids were done by MS^2^ and MS^3^ experiments with the orbitrap mass analyzer. The analysis was done with positive ionization, the orbitrap full scan analysis was run with a resolution of 120,000, while MS^2^ and MS^3^ experiments were run at 15,000. Identification was obtained by first running a full scan in the mass range m/z 250–1,500, followed by data dependent MS^2^ (ddMS^2^) where the most intense ions were selected for HCD fragmentation with stepped collision energy (25, 30, 35%). Based on the results from the first fragmentation the ions could be sent for a second MS^2^ analysis if the result showed that the molecule was a phosphocholine (based on m/z 184 corresponding to the head group) for full identification by CID fragmentation. If the first ddMS^2^ showed that the molecule was a triacylglycerol (by neutral loss of a fatty acid) the ions were sent for MS^3^ analysis by CID fragmentation for full identification. All other lipids were identified after the first ddMS^2^ fragmentation. To identify as many lipids as possible in the samples 5 consecutive injections of a pooled sample were analysed with MS^n^ fragmentation. In the first injection the most intense signals are fragmented for identification, thereafter, put on an exclusion list for injection 2 so that signals with lower intensity are fragmented and identified in the second injection. The process is repeated for all 5 injections to ensure identification of all lipids with significant signal intensity. The samples were analyzed in full scan with a resolution of 120,000, and after analysis the results were aligned with the results from the MS^2^ and MS^3^ experiment for identification. Lipid identification and relative quantification were done with the software LipidSearch from Thermo. LipidSearch data were filtered to only include Grade A and B identifications of phospholipids, in addition a manual inspection of identifications was performed to have as high degree of identification certainty as possible. All details for the LC-MS method can be found in the Supplementary Material.

### Liposomes preparation

The lipid film from *E*. *coli* Δ*tolA* culture lipid isolate was prepared by removing the solvent under vacuo at 45°C on a rotavapor with final step of holding at vacuum of 55 hPa at room temperature for 3 h. The lipid film was then hydrated with TRIS buffer. Before extrusion through 100 nm polycarbonate membrane filters (Millipore) the liposome dispersions were subjected to 5 min of sonication cycles (5 s impulse followed by 10 s hold) at room temperature using probe sonicator. Size and zeta-potential of resulting liposomes were analyzed by zetasizer (see below).

### Size and zeta potential measurements

Samples of isolated bEVs and freshly extruded liposomes prepared from lipid isolate dispersed in TRIS buffer were subjected to size and zeta potentials measurements using a Malvern Zetasizer Nano ZS (Malvern, Oxford, United Kingdom) instrument. In addition to size measurements, the polydispersity index (PDI) was estimated to assess population homogeneity. For comparison zeta-potential of the bacterial cells of the presented strains were determined by growing a 10 mL mini-prep of bacterial culture in LB media for 6 h and measuring the zeta-potential by diluting the samples in TRIS buffer. All zeta-potential measurements were performed using the disposable folded capillary cells (DTS1070). All samples were measured in triplicates at 25°C.

### Electron microscopy

For negative stain electron microscopy freshly carbon-coated 400 mesh grids were used. The grids were absorbed on the droplets of isolated bEVs for 5 min, washed with double distilled water and stained with 1:9 mixture of 3% uranyl acetate and 2% methylcellulose for 2 min. The grids with the sample were picked up with loops, excess uranyl acetate was removed with filter paper, and the loops with the grids were dried on the loop holder for 10 min prior the imaging. The micrographs were obtained with HT7800 Series transmission electron microscope (Hitachi High-Tech Corp., Tokyo, Japan) operating at accelerated voltage of 100 kV coupled with Morada camera.

### SPR assays

bEVs isolates resuspended in TRIS buffer at 0.5 mg/mL protein concentration were used as is without additional treatment. It was not possible to keep track on the final concentration of liposomes prepared from lipid isolate. For this sample optimal dilution was empirically determined by monitoring coverage of the chip. The SPR experiments were performed as was described in (Rainsford et al., 2022; Jakubec et al., 2023). Briefly, the analysis was carried out with a T200 Biacore instrument (GE Healthcare, Chicago, Ill, United States), at 25°C. Samples of bEVs or liposomes were immobilized on the surface of L1 chip (Cytiva, Marlborough, MA, United States) at 2 μL/min flow rate for 2,400 s. Chip coverage was assessed by injection of 0.5 mg/mL of BSA at 30 μL/min flow rate for 60 s. Change in response units (RU) of less than 400 was considered as sufficient coverage of the chip. The peptides were tested at increasing range of concentrations (from 4 to 128 µM for c-WRWRWR, c-WWWKKK and c-WWWRRR or 24–768 µM for c-WKWKWK and c-LWwNKr) in 10 mM HEPES buffer (with 150 mM NaCl). The peptides were injected over immobilized samples at 15 μL/min flow rate for 200 s followed by 400 s of dissociation phase. After each injection, the surface of immobilized vesicles was stabilized by three subsequent injections of 10 mM NaOH at 30 μL/min flow rate for 30 s each. Each run was concluded with cleaning of the chip surface by subsequently injecting 10 mM CHAPS, 40 mM octyl-β-D-glucopyranoside and 30% ethanol for 1 min at 30 μL/min flow rate. The control channel of the chip was treated the same way, except only HEPES buffer was injected instead of peptide solutions. The resulting data sets were processed and analyzed by in-house developed MATLAB scripts (MATLAB R2020a; available at: https://github.com/MarJakubec). K_D_ was obtained using steady-state fit (Eq. 1) and k_off_ rate constant was determined using formalism suggested by Figueira et al., 2017 (Eqs 2, 3). The association k_on_ rate was calculated from obtained K_D_ and k_off_ values using Eq. 4.

$$RU=\frac{{RU}_{\mathrm{MAX}}}{1+\frac{K_{D}}{\left[P\right]}}+off$$ (1)

$$S_{L}\left(t\right)=\alpha e^{-k_{off},\alpha^{t}}+\beta e^{{-k}_{off},\beta^{t}}+S_{L,r}$$ (2)

$$k_{off}=\frac{\alpha k_{off},\alpha +\beta k_{off},\beta }{\alpha +\beta }$$ (3)

$$K_{D}=\frac{k_{off}}{k_{on}}$$ (4)

where RU is measured SPR response; RU_MAX_ is RU response at saturation; [P] is peptide concentration, and *off* is the offset of the response; S_L_ is the linearised ratio of responses of the solute, which is plotted against the time of dissociation; α and β are individual populations, and S_L,r_ is the retained solute fraction.

### ^31^P NMR spectroscopy

NMR spectra were acquired using a Bruker 400 MHz Avance III HD spectrometer equipped with an RT SmartProbe. Lipid isolates from bacterial membranes were dissolved in 500 µL of CUBO solvent (800 mM guanidine chloride dissolved in 3:1 mixture of dimethylformamide (DMF) and trimethylamine). For lipid quantification trimethyl phosphate (TMP) standard was added to the samples at the concentration of 0.7 µM. All experiments were acquired at 298 K in 5 mm NMR tubes. Spectra were processed in TopSpin 4.1.4 (Bruker, Billerica, MA, United States).

## Data Availability

The original contributions presented in the study are included in the article/Supplementary Material, further inquiries can be directed to the corresponding author.

## Abbreviations

AMPs, antimicrobial peptides; AMR, antimicrobial resistance; bEVs, bacterial extracellular vesicles; DMPC, dimyristoylphosphatidylcholine; ESKAPE, *Enterococcus faecium*, *Staphylococcus aureus*, *Klebsiella pneumoniae*, *Acinetobacter baumannii*, *Pseudomonas aeruginosa*, and *Enterobacter* spp.; LPS, lipopolysaccharides; MIC, minimum inhibitory concentration; MS, mass spectrometry; NMR, nuclear magnetic resonance spectroscopy; PLs, phospholipids; SPR, surface plasmon resonance.
