# Supplementary material for: Bacterial extracellular vesicles: towards realistic models for bacterial membranes in molecular interaction studies by surface plasmon resonance
Source: Front Mol Biosci. 2023 Dec 13;10:1277963. doi: 10.3389/fmolb.2023.1277963 (PMC10751319; doi:10.3389/fmolb.2023.1277963)
Supplement: Supplementary file 1 [file DataSheet1.PDF]

SUPPLEMENTARY TABLES AND FIGURES

Table S1

Table S1. Characterization of size and zeta potential of the isolated bEVs and liposomes prepared from *E. coli* Δ*tolA* lipid isolate.

Sample	Size, nm	PDI	Zeta potential, mV	
			bEVs	Cell
Liposomes	139.9 ± 1.6	0.299 ± 0.01	− 17.8 ± 0.5	
bEVs:				
E. coli Δ*tolA*	82.7 ± 1.3	0.246 ± 0.01	− 9.4 ± 0.8	− 6.07 ± 1.4
K. pneumoniae	194.8 ± 1.0	0.333 ± 0.01	− 9.9 ± 0.3	− 5.39 ± 0.8
A. baumannii	175.0 ± 1.3	0.240 ± 0.00	− 14.4 ± 0.6	− 9.20 ± 0.5
P. aeruginosa	143.3 ± 1.1	0.103 ± 0.01	− 3.9 ± 0.6	− 4.71 ± 1.6

Table S2

Table S2. Fatty acid composition within PE class of lipids analyzed by mass spectrometry

Strains	PE fatty acid composition	Lipid isolate		PE fatty acid composition	bEVs	
		Relative abundance	st. dev.		Relative abundance	st. dev.
E. coli ΔtolA	PE 16:0_16:1	0.19	0.04	PE 16:0_16:1	0.27	0.09
	PE 16:1_18:1	0.16	0.00	PE 16:1_18:1	0.12	0.06
	PE 16:0_18:1	0.12	0.01	PE 16:0_18:1	0.11	0.01
	PE 18:1_18:1	0.11	0.04	PE 16:0_17:1	0.08	0.02
	PE 16:0_17:1	0.09	0.00	PE 18:1_18:1	0.06	0.01
	PE 18:4_16:0	0.03	0.00	PE 18:4_16:0	0.05	0.00
	PE 17:1_18:1	0.03	0.03	PE 16:1_16:1	0.04	0.00
	PE 16:1_16:1	0.02	0.00	PE 14:0_16:0	0.03	0.00
	PE 20:4_16:1	0.02	0.00	PE 17:1_18:1	0.02	0.00
	PE 16:1_17:1	0.02	0.00	PE 20:4_16:1	0.02	0.00
	PE 14:0_16:0	0.02	0.00	PE 18:3_16:1	0.02	0.01
K. pneumoniae	PE 16:0_17:1	0.29	0.04	PE 16:0_16:1	0.12	0.01
	PE 16:0_19:1	0.16	0.00	PE 14:0_16:0	0.12	0.03
	PE 14:0_16:0	0.15	0.01	PE 16:0_17:1	0.12	0.00
	PE 19:1_17:1	0.10	0.00	PE 16:0_18:1	0.12	0.01
	PE 19:1_19:1	0.05	0.01	PE 16:0_19:1	0.08	0.00
	PE 14:0_17:1	0.05	0.00	PE 16:0_16:0	0.04	0.00
	PE 17:1_17:1	0.03	0.00	PE 16:1_18:1	0.04	0.01
	PE 16:0_16:0	0.03	0.01	PE 14:0_17:1	0.03	0.00
	PE 14:0_14:0	0.01	0.01	PE 16:0_18:1	0.02	0.01
A. baumannii	PE 16:0_18:1	0.28	0.03	PE 16:0_18:1	0.35	0.03
	PE 18:1_18:1	0.16	0.00	PE 16:1_18:1	0.25	0.00
	PE 16:1_18:1	0.10	0.01	PE 18:0_18:1	0.04	0.00
	PE 14:0_18:1	0.09	0.00	PE 18:1_18:0	0.04	0.00
	PE 15:0_18:1	0.06	0.00	PE 18:1_18:1	0.04	0.01
	PE 16:0_19:1	0.06	0.00	PE 16:0_16:1	0.04	0.00
	PE 16:0_16:0	0.05	0.00	PE 16:0_16:0	0.02	0.00
	PE 17:1_18:1	0.02	0.00	PE 16:1_16:1	0.02	0.00
	PE 18:4_19:0	0.02	0.00	PE 16:0_19:1	0.01	0.00
P. aeruginosa	PE 16:0_18:1	0.36	0.07	PE 16:0_18:1	0.44	0.01
	PE 16:0_19:1	0.13	0.00	PE 16:1_18:1	0.11	0.03
	PE 16:1_18:1	0.09	0.00	PE 16:0_16:1	0.06	0.00
	PE 17:1_18:1	0.04	0.00	PE 16:0_16:0	0.06	0.00
	PE 15:0_18:1	0.04	0.00	PE 16:0_18:0	0.04	0.00
	PE 16:1_18:1	0.04	0.01	PE 16:0_16:1	0.02	0.00
	PE 16:0_16:0	0.04	0.00	PE 18:1_18:1	0.02	0.00
	PE 14:0_18:1	0.03	0.01	PE 16:1_18:1	0.02	0.01
	PE 18:1_18:1	0.03	0.01	PE 16:0_19:1	0.01	0.00

Table S3

Table S3. Dissociation constant K_D determined by SPR

AMP	K_D, μM				
	Liposomes	*E. coli ΔtolA*	*K. pneumoniae*	*A. baumannii*	*P. aeruginosa*
c-LWwNKr	966.5 ± 170.1	406.4 ± 38.1	1075.2 ± 49.0	857.8 ± 61.4	1036.5 ± 269.7
c-WKWKWK	244.4 ± 39.5	380.9 ± 22.6	693.7 ± 37.3	389.0 ± 32.5	749.3 ± 117.3
c-WRWRWR	74.5 ± 3.3	73.0 ± 7.3	81.1 ± 2.2	113.6 ± 5.2	118.5 ± 3.5
c-WWWKKK	28.3 ± 4.2	56.5 ± 1.0	21.0 ± 0.7	100.2 ± 13.1	100.0 ± 4.1
c-WWWRRR	47.8 ± 5.2	27.0 ± 1.6	35.8 ± 1.3	48.7 ± 2.7	59.0 ± 2.6

Table S4

Table S4. k_{off} rate constant determined by SPR

AMP	k_{off}, s^{-1}				
	Liposomes	*E. coli ΔtolA*	*K. pneumoniae*	*A. baumannii*	*P. aeruginosa*
c-LWwNKr	1.67 ± 0.45	1.42 ± 0.27	1.75 ± 0.55	1.72 ± 0.45	1.78 ± 0.54
c-WKWKWK	0.95 ± 0.20	1.42 ± 0.16	1.51 ± 0.54	0.82 ± 0.11	1.52 ± 0.37
c-WRWRWR	0.57 ± 0.09	0.74 ± 0.10	0.59 ± 0.10	0.64 ± 0.13	0.42 ± 0.07
c-WWWKKK	0.21 ± 0.01	0.62 ± 0.05	0.37 ± 0.03	0.63 ± 0.12	0.62 ± 0.07
c-WWWRRR	0.51 ± 0.08	0.18 ± 0.02	0.28 ± 0.01	0.40 ± 0.06	0.37 ± 0.04

Table S5

Table S5. Calculated k_{on} rate constant

AMP	k_{on}, $\mu M^{-1}\cdot s^{-1}$				
	Liposomes	*E. coli ΔtolA*	*K. pneumoniae*	*A. baumannii*	*P. aeruginosa*
c-LWwNKr	0.002 ± 0.001	0.003 ± 0.001	0.002 ± 0.001	0.002 ± 0.001	0.002 ± 0.001
c-WKWKWK	0.004 ± 0.001	0.004 ± 0.001	0.002 ± 0.001	0.002 ± 0.001	0.002 ± 0.001
c-WRWRWR	0.008 ± 0.001	0.010 ± 0.001	0.007 ± 0.001	0.006 ± 0.001	0.004 ± 0.001
c-WWWKKK	0.007 ± 0.001	0.011 ± 0.001	0.018 ± 0.001	0.006 ± 0.002	0.006 ± 0.001
c-WWWRRR	0.011 ± 0.001	0.007 ± 0.001	0.008 ± 0.001	0.008 ± 0.002	0.006 ± 0.001

Table S6

Table S6. Summary of statistical analysis of SPR data with T-test

AMP	p-value	K_D				k_{off}				k_{on}			
		LI	EC	KP	AB	LI	EC	KP	AB	LI	EC	KP	AB
c-LWwNKr	EC	0.00	–	–	–	0.22	–	–	–	0.01	–	–	–
	KP	0.17	0.00	–	–	0.43	0.20	–	–	0.40	0.01	–	–
	AB	0.18	0.00	0.00	–	0.45	0.19	0.47	–	0.32	0.02	0.24	
	PA	0.36	0.01	0.41	0.16	0.40	0.18	0.47	0.45	0.43	0.00	0.43	0.22
c-WKWKWK	EC	0.00	–	–	–	0.02	–	–	–	0.38	–	–	–
	KP	0.00	0.00	–	–	0.05	0.40	–	–	0.01	0.02	–	–
	AB	0.00	0.37	0.00		0.19	0,00	0.05	–	0.00	0.01	0.48	–
	PA	0.00	0.00	0.24	0.00	0,03	0.26	0.43	0.01	0.00	0.01	0.46	0.46
c-WRWRWR	EC	0.38	–	–	–	0.05	–	–	–	0.03	–	–	–
	KP	0.02	0.07	–	–	0.41	0.07	–	–	0.37	0.02	–	–
	AB	0.00	0.00	0.00	–	0.22	0.18	0.29	–	0.07	0.00	0.01	–
	PA	0.00	0.00	0.00	0.24	0.04	0.01	0.04	0.03	0.01	0.00	0.01	0.02
c-WWWKKK	EC	0.00	–	–	–	0.00	–	–	–	0.01	–	–	–
	KP	0.02	0.00	–	–	0.00	0.00	–	–	0.00	0.00	–	–
	AB	0.00	0.00	0.00	–	0.00	0.41	0.01	–	0.26	0.01	0.00	–
	PA	0.00	0.00	0.00	0.49	0.00	0.48	0.00	0.43	0.13	0.00	0.00	0.41
c-WRWRWR	EC	0.00	–	–	–	0.00	–	–	–	0.03	–	–	–
	KP	0.01	0.00	–	–	0.00	0.00	–	–	0.06	0.05	–	–
	AB	0.40	0.00	0.00	–	0.01	0.00	0.01	–	0.06	0.05	0.23	–
	PA	0.01	0.00	0.00	0.00	0.02	0.00	0.01	0.03	0.02	0.33	0.03	0.00

Here: LI – liposomes prepared from *E. coli* Δ*tolA* lipid isolate, EC – *E. coli* Δ*tolA*, KP – *K. pneumoniae*, AB – *A. baumannii*, PA – *P. aeruginosa*. p-value ≥ 0.05 is highlighted in red.

Figure S1

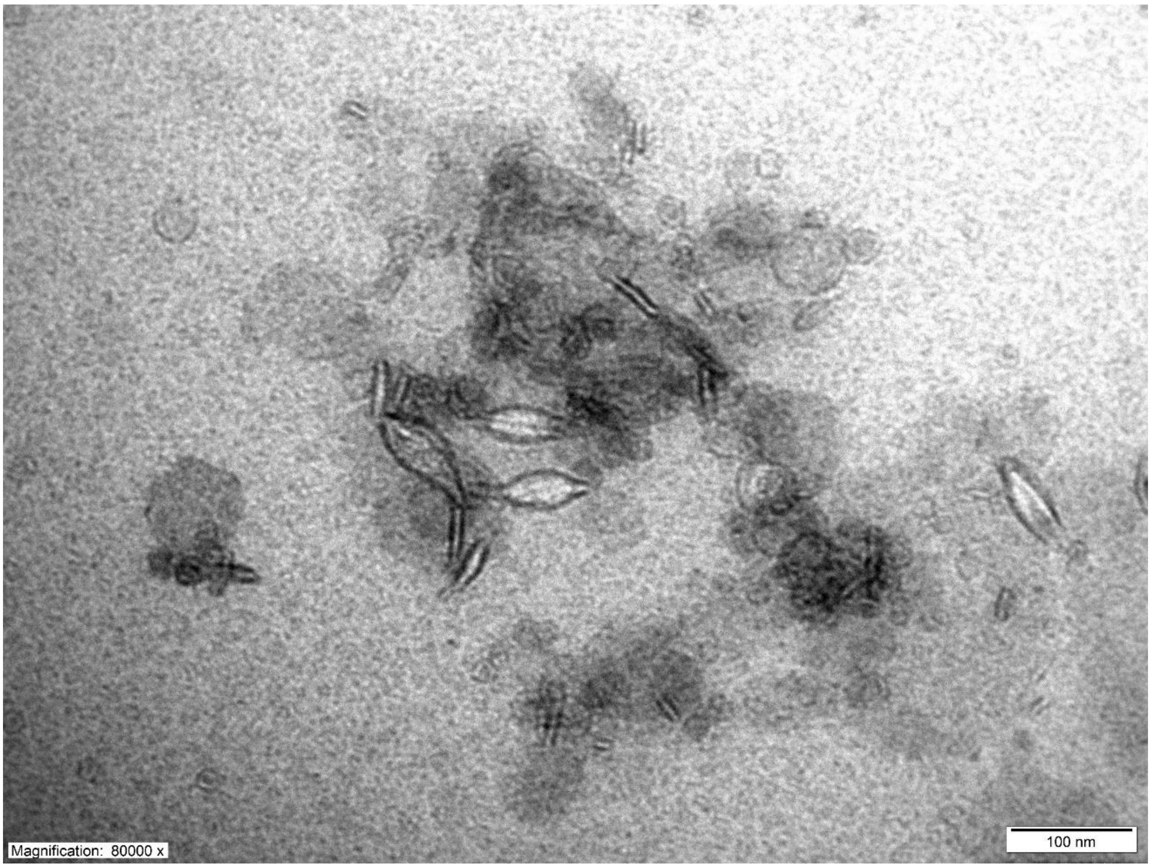

Figure S1. TEM micrograph of bEVs secreted by *E. coli* NR698 strain at 80,000x magnification. 100 nm scalebar is presented at the bottom right corner of the micrograph.

Figure S2

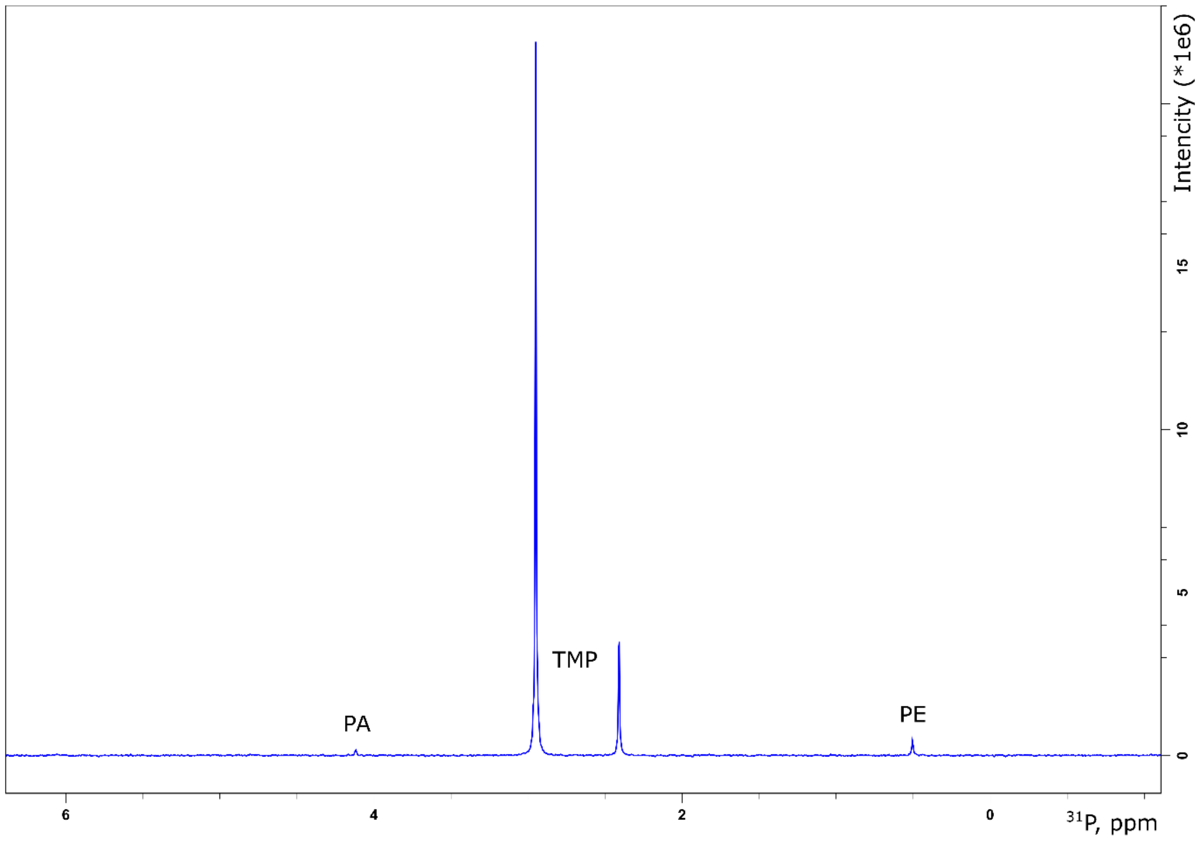

Figure S2. ^{31}P NMR spectra of lipid isolates prepared from LB media components. Here PE – phosphatidylethanolamine; PA – phosphatidic acid; TMP – trimethyl phosphate (standard added).

```
Thermo Scientific SII for Xcalibur Method

---- Overview ----
Name: New Instrument Method
Comment:
Run time: 32.500 [min]
Instrument: Vanquish_H on thermo-jr7hgt84
Description:
---- Script ----
initial      Instrument Setup
             ColumnComp.PrehtLeft.ReadyTempDelta: 1.00 [°C]
             ColumnComp.PrehtLeft.TempCtrl: On
             ColumnComp.PrehtLeft.Temperature.Nominal: 60.00 [°C]
             ColumnComp.PrehtLeft.EquilibrationTime: 1.0 [min]
             ColumnComp.CC.Mode: StillAir
             ColumnComp.CC.ReadyTempDelta: 0.50 [°C]
             ColumnComp.CC.TempCtrl: On
             ColumnComp.CC.Temperature.Nominal: 60.00 [°C]
             ColumnComp.CC.EquilibrationTime: 1.0 [min]
             ColumnComp.Column_B.ActiveColumn: No
             ColumnComp.Column_B.SystemPressure: "Pump"
             ColumnComp.Column_D.ActiveColumn: Yes
             SamplerModule.Sampler.PunctureOffset: 0 [µm]
             SamplerModule.Sampler.WashSpeed: 20.0 [µl/s]
             SamplerModule.Sampler.InjectWashMode: Both
             SamplerModule.Sampler.WashTime: 5.0 [s]
             SamplerModule.Sampler.DispenseSpeed: 5.000 [µl/s]
             SamplerModule.Sampler.DrawSpeed: 5.000 [µl/s]
             SamplerModule.Sampler.Pump: "Pump"
             SamplerModule.TempCtrl: On
             SamplerModule.Temperature.Nominal: 25.0 [°C]
             PumpModule.Pump.%B_Selector: %B1
             PumpModule.Pump.%A_Selector: %A3
             PumpModule.Pump.%A1_Equate: "Water 1 mM NH4FA 0.01 FA"
             PumpModule.Pump.%A2_Equate: "Water 0.1% FA"
             PumpModule.Pump.%A3_Equate: "50% ACN 1 mM NH4FA 0.01 FA"
             PumpModule.Pump.%B1_Equate: "50/50 IPA/MeCN 1 mM NH4FA 0.01 FA"
             PumpModule.Pump.%B2_Equate: "MeCN 0.1% FA"
             PumpModule.Pump.%B3_Equate: "95% ACN 1 mM NH4FA 0.01 FA"
             PumpModule.Pump.Pressure.LowerLimit: 0 [bar]
             PumpModule.Pump.Pressure.UpperLimit: 1517 [bar]
             PumpModule.Pump.MaximumFlowRampUp: 6.00 [ml/min²]
             PumpModule.Pump.MaximumFlowRampDown: 6.00 [ml/min²]
             ColumnComp.LowerValve.CurrentPosition: 6_1
             ColumnComp.UpperValve.CurrentPosition: 6_1
-2.500 [min] Equilibration
             PumpModule.Pump.Flow.Nominal: 0.600 [ml/min]
             PumpModule.Pump.%B.Value: 30.0 [%]
             PumpModule.Pump.Curve: 5
0.000 [min]
```

```
Thermo Scientific SII for Xcalibur Method

            PumpModule.Pump.Flow.Nominal: 0.600 [ml/min]
            PumpModule.Pump.%B.Value: 30.0 [%]
            PumpModule.Pump.Curve: 5
0.000 [min] Inject Preparation
            Wait ColumnComp.Ready And SamplerModule.Sampler.Ready And PumpModule.Pump.Ready
0.000 [min] Inject
            SamplerModule.Sampler.Inject
0.000 [min] Start Run
            ColumnComp.CC_Temp.AcqOn
            ColumnComp.PrehtLeft_Temp.AcqOn
            PumpModule.Pump.Pump_Pressure.AcqOn
0.000 [min] Run
            PumpModule.Pump.Flow.Nominal: 0.600 [ml/min]
            PumpModule.Pump.%B.Value: 30.0 [%]
            PumpModule.Pump.Curve: 5
20.000 [min]
            PumpModule.Pump.Flow.Nominal: 0.600 [ml/min]
            PumpModule.Pump.%B.Value: 75.0 [%]
            PumpModule.Pump.Curve: 5
25.000 [min]
            PumpModule.Pump.Flow.Nominal: 0.600 [ml/min]
            PumpModule.Pump.%B.Value: 95.0 [%]
            PumpModule.Pump.Curve: 5
30.000 [min] Stop Run
            ColumnComp.CC_Temp.AcqOff
            ColumnComp.PrehtLeft_Temp.AcqOff
            PumpModule.Pump.Pump_Pressure.AcqOff
```

Method Summary

Method Settings

Application Mode: **Small Molecule**
Method Duration (min): **30**

Global Parameters

Ion Source

Use Ion Source Settings from Tune: **True**
FAIMS Mode: **Not Installed**

MS Global Settings

Infusion Mode: **Liquid Chromatography**
Expected LC Peak Width (s): **3**
Advanced Peak Determination: **False**
Mild Trapping: **False**
Default Charge State: **1**
Enable Xcalibur AcquireX method modifications: **True**
Internal Mass Calibration: **EASY-IC™**

Divert Valve A

Time (min)	Position
0	1-2

Experiment#1 [AcquireX lipid characterization HCD-CID-MS3]

Start Time (min): **0**
End Time (min): **30**
Cycle Time (sec): **1.5**

Master Scan:

MS OT

Detector Type: **Orbitrap**
Orbitrap Resolution: **120000**
Use Quadrupole Isolation: **True**
Scan Range (m/z): **250-1500**
RF Lens (%): **40**
AGC Target: **Standard**
Maximum Injection Time Mode: **Custom**
Maximum Injection Time (ms): **50**
Microscans: **1**
Data Type: **Profile**
Polarity: **Positive**
Source Fragmentation: **Disabled**
Use EASY-IC™: **True**
Scan Description:

Filters:

Intensity

Filter Type: **Intensity Threshold**
Intensity Threshold: **1.0e5**

Dynamic Exclusion

Exclude after n times: **1**
Exclusion duration (s): **2**
Mass Tolerance: **ppm**
Low: **10**
High: **10**
Exclude Isotopes: **True**

Targeted Mass Exclusion

Mass List

Mass List Type: **m/z**
Time Mode: **Start/End Time**
Include Intensity Threshold: **True**
Add Mass List Targets Determined by Xcalibur AcquireX: **True**

Compound	m/z	t start (min)	t stop (min)	Intensity Threshold
	524.265	0	30	1E+20

Exclusion mass width: **ppm**
Low: **10**

High: **10**

Data Dependent

Data Dependent Mode: **Cycle Time**
Time between Master Scans (sec): **1.5**

Scan Event Type 1:

Targeted Mass

Mass List

Mass List Type: **m/z**
Time Mode: **Start/End Time**
Include Intensity Threshold: **True**
Add Mass List Targets Determined by Xcalibur AcquireX: **True**

Compound	m/z	t start (min)	t stop (min)	Intensity Threshold
	524.265	0	30	0

Mass Tolerance: **ppm**
Low: **10**
High: **10**
Set Collision Energy per Compound: **False**
Perform dependent scan on most intense ion if no targets are found: **True**
Use Group IDs: **False**

Scan:

ddMS^2 OT HCD

Isolation Mode: **Quadrupole**
Isolation Window (m/z): **1.5**
Isolation Offset: **Off**
Activation Type: **HCD**
Collision Energy Mode: **Stepped**
HCD Collision Energy Type: **Normalized**
HCD Collision Energies (%): **25,30,35**
Detector Type: **Orbitrap**
Orbitrap Resolution: **15000**
Scan Range Mode: **Define First Mass**
First Mass (m/z): **140**
AGC Target: **Standard**
Maximum Injection Time Mode: **Custom**

Maximum Injection Time (ms): **50**
Microscans: **1**
Data Type: **Profile**
Use EASY-IC™: **True**
Scan Description:

Data Dependent

Data Dependent Mode: **Scans Per Outcome**

Scan Event Type 1:

Targeted Mass Trigger

Mass List

Mass List Type: **m/z**

Compound	m/z
	184.0733

Mass Tolerance: **ppm**
Low: **10**
High: **10**
Use Group IDs: **False**
Trigger Only with Detection of at Least N Ions from the List: **False**
Only Ion(s) Within Top N Most Intense: **True**
n :: **3**
Only Ion(s) Above the Threshold (Relative Intensity, %): **False**
Trigger Type: **Continue Trigger**

Intensity

Filter Type: **Intensity Threshold**
Intensity Threshold: **5.0e4**

Scan:

ddMS^2 OT CID

MS^n Level: **2**
Scan Priority: **1**
Isolation Mode: **Quadrupole**
Isolation Window (m/z): **2**
Isolation Offset: **Off**

Activation Type: **CID**
Collision Energy Mode: **Fixed**
CID Collision Energy (%): **32**
CID Activation Time (ms): **10**
Activation Q: **0.25**
Multistage Activation: **False**
Detector Type: **Orbitrap**
Orbitrap Resolution: **15000**
Scan Range Mode: **Auto**
AGC Target: **Standard**
Maximum Injection Time Mode: **Custom**
Maximum Injection Time (ms): **50**
Microscans: **1**
Data Type: **Profile**
Use EASY-IC™: **False**
Scan Description:
Number of Dependent Scans: **1**

Scan Event Type 2:

Targeted Loss Trigger

Mass List

Mass List Type: **m/z**

Compound	m/z
	217.2042
	215.1885
	245.2355
	243.2198
	273.2668
	271.2511
	269.2355
	287.2824
	285.2668
	301.2981
	299.2824
	297.2668

	295.2511
	315.3137
	329.3294
	327.3137
	325.2981
	323.2824
	321.2668
	319.2511
	343.345
	357.3607
	355.345
	353.3294
	351.3137
	349.2981
	347.2824
	345.2668
	371.3763
	385.392
	383.3763
	413.4233

Mass Tolerance: **ppm**
Low: **10**
High: **10**
Trigger Only with Detection of at Least N Ions from the List: **False**
Only Ion(s) Within Top N Most Intense: **True**
n :: **3**
Only Ion(s) Above the Threshold (Relative Intensity, %): **False**
Trigger Only with Detection of Correct Charge State of the Product Ion: **False**
Ignore Charge State Requirement for Unassigned Ions: **False**
Trigger Type: **Continue Trigger**

Precursor Ion Exclusion

Exclusion mass width: **m/z**
Low: **0.5**
High: **4**

Intensity

Filter Type: **Intensity Threshold**
Intensity Threshold: **5.0e4**

Scan:

ddMS^3 OT CID

MS^n Level: **3**
Scan Priority: **1**
MS Isolation Window (m/z): **1.5**
MS2 Isolation Window (m/z): **2**
Isolation Offset: **Off**
Activation Type: **CID**
Collision Energy Mode: **Fixed**
CID Collision Energy (%): **35**
CID Activation Time (ms): **10**
Activation Q: **0.25**
Multistage Activation: **False**
Detector Type: **Orbitrap**
Orbitrap Resolution: **15000**
Scan Range Mode: **Auto**
AGC Target: **Standard**
Maximum Injection Time Mode: **Custom**
Maximum Injection Time (ms): **65**
Microscans: **1**
Data Type: **Profile**
Use EASY-IC™: **False**
Scan Description:
Number of Dependent Scans: **3**
