# Supplementary figures and images for: Bacterial extracellular vesicles: towards realistic models for bacterial membranes in molecular interaction studies by surface plasmon resonance
Source: Front Mol Biosci. 2023 Dec 13;10:1277963. doi: 10.3389/fmolb.2023.1277963 (PMC10751319; doi:10.3389/fmolb.2023.1277963)

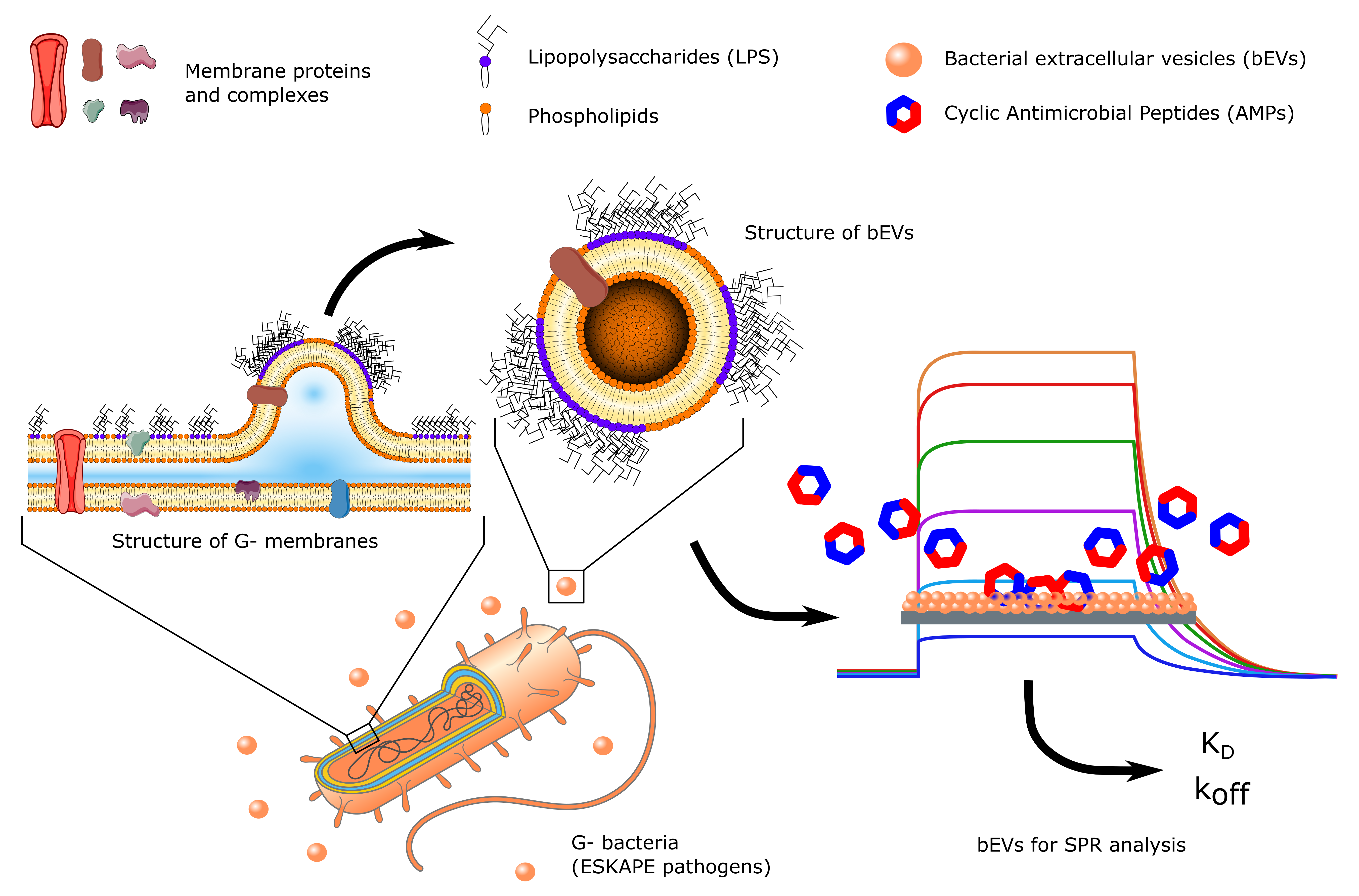

Supplement: Supplementary file 2 [file Image1.PNG]
